# Potentially Bio-Accessible Metabolites from an Extract of *Cornus mas* Fruit after Gastrointestinal Digestion In Vitro and Gut Microbiota Ex Vivo Treatment

**DOI:** 10.3390/nu14112287

**Published:** 2022-05-30

**Authors:** Agata Olędzka, Katarzyna Cichocka, Konrad Woliński, Matthias F. Melzig, Monika E. Czerwińska

**Affiliations:** 1 Student Scientific Association “Farmakon”, Department of Biochemistry and Pharmacogenomics, Medical University of Warsaw, 1 Banacha Street, 02-097 Warsaw, Poland; agata.bobinska2997@gmail.com (A.O.); katarzyna.cichocka.kc@gmail.com (K.C.); 2 Polish Academy of Sciences Botanical Garden, Centre for Biological Diversity Conservation in Powsin, 2 Prawdziwka Street, 02-973 Warsaw, Poland; k.wolinski@obpan.pl; 3 Institute of Pharmacy, Freie Universitaet Berlin, 2+4 Koenigin-Luise Street, 14195 Berlin, Germany; matthias.melzig@fu-berlin.de; 4 Department of Biochemistry and Pharmacogenomics, Faculty of Pharmacy, Medical University of Warsaw, 1 Banacha Street, 02-097 Warsaw, Poland; 5 Centre for Preclinical Research, Medical University of Warsaw, 1B Banacha Street, 02-097 Warsaw, Poland

**Keywords:** cornelian cherries, cornuside, iridoids, gut microbiota, metabolism

## Abstract

Targeting pancreatic lipase and α-amylase by digestion-derived fractions of ethanolic-aqueous (60%, *v*/*v*) extract from *Cornus mas* fruit (CM) in relation to the control and prevention of metabolic disorders, including diabetes, was the first purpose of the present study. Taking into consideration the significance of bio-accessibility of compounds, we attempted to identify metabolites of CM after gastrointestinal digestion in vitro, as well as their kinetic changes upon gut microbiota treatment. The digestion of extract was simulated with digestive enzymes in vitro and human gut microbiota ex vivo (1 h, 3 h, 6 h, 24 h), followed by chromatographic analysis using the UHPLC-DAD-MS*^n^* method. The effect of fractions from gastrointestinal digestion in vitro on the activity of pancreatic lipase and α-amylase was studied with fluorescence-based assays. The gastric and intestinal fractions obtained after in vitro digestion of CM inhibited pancreatic lipase and α-amylase. Loganic acid as the main constituent of the extract was digested in the experimental conditions in contrast to cornuside. It was found in most analytes such as salivary, gastric, intestinal, and even colon (fecal slurry, FS) fractions. In all fractions, kaempferol hexoside and reduced forms of kaempferol, such as aromadendrin, and benzoic acid were assigned. The signals of tannins were detected in all fractions. Cornusiin A was tentatively assigned in the gastric fraction. The metabolites originating from kinetic analytes have been classified mainly as phenolic acids, hydrolyzable tannins, and flavonoids. Phenolic acids (protocatechuic acid, gallic acid), tannins (digalloylglucose, tri-*O*-galloyl-*β*-D-glucose), and flavonoids (aromadendrin, dihydroquercetin) were detected in the late phases of digestion in fecal slurry suspension. Cornuside was found in FS analyte after 3 h incubation. It was not detected in the samples after 6 and 24 h incubation with FS. In conclusion, cornuside, aromadendrin, and phenolic acids may be potentially bio-accessible compounds of CM. The presence of plants’ secondary metabolites in the intestinal fractions allows us to indicate them as responsible for decreasing glucose and lipid absorption.

## 1. Introduction

Changing life facilities leading to a decrease in physical activity in addition to an increased intake of energy-dense food results in obesity, which is dramatically increasing. The prevalence of obesity has tripled since 1975, according to the World Health Organization. In 2016, there were more than 39% (1.9 billion) overweight adults aged over 18 years [1]. Obesity is considered a risk factor for several chronic disorders, including cardiovascular diseases, diabetes complicated by blindness, limb amputations, kidney failure, several cancers, and musculoskeletal disorders [1,2].

Promoting healthy, plant-based diets, reducing the fat, sugar, and salt content in processed foods, as well as increasing physical activity are key support in obesity prevention. The booster of these supportive fundamentals is an increased consumption of fruit and vegetables, taking into consideration that some of them inhibit the uptake of glucose and fructose [3] or lipids [4]. Increasing attention has been paid to the significance of the gut microbiome in the development of obesity-related complications recently [2]. However, the bidirectional influence of nutrients and gut microbiota determines health and beneficial effects for the host. The arisen questions concern the effect of plant materials on gut microbiota and its effect on metabolic changes of phytochemicals and their bio-accessibility. The stability of chemicals in the gastrointestinal tract determines the first step of pharmacokinetics such as absorption. The bioavailability of natural compounds occurring in plant materials, food, and their preparation is particularly in the field of interest when their health beneficial effects require justification or molecular background.

Fruits of cornelian cherry (*Cornus mas* L., Cornaceae) are traditionally known to support the therapy for a plethora of ailments, such as diarrhea, fever, and gastrointestinal problems, and to enhance kidney and liver functions. Currently, it is mostly used in the food industry for the production of jams, juices, and liqueurs [5,6,7].

*Cornus mas* is a source of iridoids, such as loganic acid and cornuside, as well as anthocyanins, such as 3-*O*-rutinoside and 3-*O*-glucoside of pelargonidin, delphinidin 3-*O*-rutinoside, as well as cyanidin 3-*O*-galactoside. It is believed that particularly anthocyanins play a part in the reduction of body weight and liver lipids without influencing food intake in rats as well as alleviating the course of diabetes [7]. Anthocyanins stimulate rodent pancreatic *β*-cells for the secretion of insulin, as well as improving glucose tolerance and insulin resistance [8]. In addition, they have shown an anti-hyperglycemic effect by inhibiting *α*-glucosidase. As a result, consumption of cornelian cherry extract, with a similar effect to glibenclamide, which is a frequently used anti-diabetic drug, was observed [9]. Moreover, in a clinical trial, it was shown that *C. mas* extract intake caused a reduction of glycosylated hemoglobin (HbA1c) and triglyceride blood levels [10]. In particular, resin-purified extract of cornelian cherry, which was characterized by a high content of iridoids and anthocyanins, reduced concentrations of triglycerides, contrary to total cholesterol and LDL, in rats treated with a high-fat diet [11]. In addition, the decrease of leptin and increase of adiponectin concentrations were noted in the study by Danielewski et al. [11].

Furthermore, the active compounds in cornelian cherry are proven to neutralize active forms of oxygen. It is considered that the antioxidant effect is relevant to preventing changes in blood cell structure, which can occur during diabetes [12]. Apart from direct sugar and lipid-lowering activity, targeting inflammatory pathways is crucial during the treatment of this disease [13]. The anti-inflammatory effect of *C. mas* extract was evaluated in various models. Cornelian cherry fruit suppresses the release of cytokines, such as interleukin IL-1*β* and IL-13, in the soft paw tissue of Wistar rats after induction of inflammation [14]. A similar effect was determined in the hypercholesterolemic rabbits—the administration of *C. mas* extract resulted in reducing the concentrations of interleukin (IL)-6 and tumor necrosis factor (TNF)-*α* [15]. Both cornuside and loganic acid have also been proven to suppress those cytokines’ activity [16,17]. In our previous study, it was shown that in human neutrophils ex vivo and colon adenocarcinoma cell line in vitro cornelian cherry significantly reduced the level of IL-8 [18], which is a chemoattractant involved in the development of inflammatory process [19]. Furthermore, according to clinical data, the consumption of *C. mas* extract resulted in a decrease of highly sensitive C-reactive protein (hsCRP) in postmenopausal women [20].

For this reason, taking into consideration anti-diabetic prerequisites of cornelian cherries displayed in previous reports, we particularly decided to investigate the inhibitory activity of cornelian cherry fractions, obtained after gastrointestinal digestion, against pancreatic lipase (PL) and *α*-amylase. Additionally, we purposed to follow the kinetic changes of secondary metabolites of the ethanolic-aqueous extract from fruits of *C. mas* treated with human gut microbiota. Our research aimed to lead us to the discovery of metabolites resistant to digestion and thus potentially bio-accessible, which may be responsible for the anti-diabetic and anti-obesity effects.

## 2. Materials and Methods

### 2.1. Reagents

Phosphate buffered saline (PBS, L0615-500), RPMI 1640 medium (L0496-500), penicillin-streptomycin (L0022-100), and fetal bovine serum (FBS, S1860-500) were purchased from Biowest (Nauillé, France). Pancoll Human (P04-601000) was from PAN-Biotech (Aidenbach, Germany). Gentamicin (15710-049) was purchased from Life Technologies (Paisley, UK). Dimethyl sulfoxide (DMSO, 113635509) was purchased from Chempur (Piekary Śląskie, Poland). Formic acid (100264) was obtained from Merck (Darmstadt, Germany). An EnzChek™ Ultra Amylase Assay Kit was purchased from (Invitrogen, Pailey, UK). Acarbose (A8980-1G), orlistat (O4139-25MG), 4-methylumbelliferyl oleate (MUO, 75164-100MG), salivary *α*-amylase (A0521-2.5KU, 170.1 units/mg), pepsin (P1725-100G, >400 U/mg protein), pancreatin from porcine pancreas (P1750-100G), as well as bile extract porcine (B8631-100G) from Sigma-Aldrich Chemie GmbH (Steinheim, Germany) were used. Tris-HCl buffer consisted of 13 mM Tris-HCl (Promega Corporation, Madison, WI USA), 150 mM NaCl, and 1.3 mM CaCl_2_ (both form POCH, Gliwice, Poland). A brain heart infusion (BHI, P-0051) was purchased from BTL (Łódź, Poland).

Acetonitrile HiPerSolv Chromanorm^®^ (20060.320) was purchased from VWR Chemicals (Radnor, PA, USA). Ethyl acetate (141-78-6) was purchased from POCH (Gliwice, Poland). HPLC grade methanol (106009) was purchased from Merck (Darmstadt, Germany).

### 2.2. Plant Material Collection

In September 2017 cornelian cherries were harvested in the Botanical Garden of Polish Academy of Sciences (Center for Biological Diversity Conservation, Poland) in Powsin (52°06′17″ N, 21°05′42″ E). A voucher specimen of twigs and leaves from *C. mas* was assigned as FW25_20160914_CM and has been deposited in the Department of Pharmacognosy and Molecular Basis of Phytotherapy (Warsaw Medical University). The plant material was identified by K. W. and M. E. C. based on the guidebook by Rutkowski [21]. Before the extract preparation, cornelian cherries were harvested and lyophilized immediately.

### 2.3. Extract Preparation

A 200 g portion of drupes (fruits with stones) of cornelian cherries were macerated three times with aqueous ethanol (60%, *v*/*v*) in a ratio of 1:10 (*m*/*v*) for 24 h each time. The collected extracts were then concentrated under reduced pressure (40 °C). The freeze extract was lyophilized in the vacuum concentrator (Telstar Cryodos 50, Terrassa, Spain) and used for further experiments.

### 2.4. Digestion Procedures In Vitro

**Figure 1:**
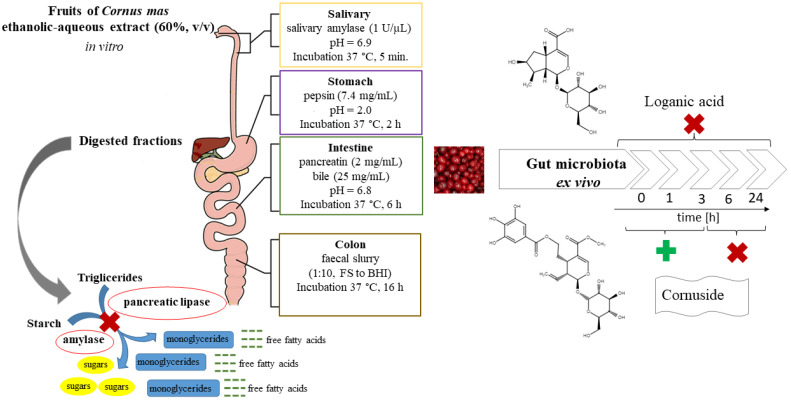
The scheme of the study.

To mimic in vitro digestion conditions, four categories were created—salivary, gastric, intestinal, and colon compartments, as it was previously described [22]. The assay was carried out in the presence (+E) or absence (−E) of standard gastrointestinal enzymes (Table 1). The pathway without digestive enzymes was conducted for control to assess if the basic digestive enzymes directly influence the changes of phytochemicals present in the CM extract. The general conditions, including the composition of gastrointestinal fractions, are presented in Table 1. The scheme of the study is displayed in Figure 1.

The fruit extract (100 mg/mL) was provided in the first compartment in the volume of 20 mL. All steps of the gastrointestinal tract simulated the mouth (CM_S, pH 6.9), gastric (CM_G. pH 2) and duodenal/intestinal (CM_I, pH 6.8) conditions. The composition of the artificial saliva was provided in Table 1. The salts were dissolved in water and incubated in a sonic bath (Sonorex Super RK 106, Bandelin, Germany) at a temperature of 30 °C for 30 min, and then mixed with the samples of fruit extract. To stimulate salivary digestion, 20 μL of salivary *α*-amylase (1 U/μL in phosphate buffer 20 mM) was added to the samples, and then they were incubated for 5 min. After each incubation, the 5 mL portions of samples were taken each time for further analysis, and 5 mL of proper solution was added to compensate for the volume gap. This procedure aimed to mimic dilutions, which take place in the gastrointestinal tract in vivo. Consequently, the volume of 5 mL of pepsin dissolved in HCl (150 mM) was added to (+E) samples, whereas in (−E) samples, the volume was adjusted with HCl solution (150 mM). The incubation was carried out for 2 h. After that, to stimulate intestinal digestion, 5 mL of the suspension of pancreatin (2 mg/mL) and bile salts (25 mg/mL) in NaHCO_3_ (0.5 M) was added and the mixture was incubated for 6 h. To provide the anaerobic conditions, the compartments were closed under a nitrogen stream and the contents were continuously mixed during incubation at 37 °C. To mimic the colon conditions (C), the residues from the first steps of digestion were incubated with 2 mL of suspension (1:10, *m*/*v*) of fecal slurry (CM_FS), adjusted with 3 mL of BHI for (+E) samples. In control samples without fecal slurries, only 5 mL of BHI was added to the (−E) samples. Finally, the samples were incubated at 37 °C in a container with GENbox anaer sachets (bioMerieux, Marcy l’Etoile, France) to provide anaerobic conditions.

The 5 mL aliquots from each compartment were extracted with ethyl acetate in a ratio of 1:10, *v*/*v*. The ethyl acetate was evaporated under reduced pressure (Rotavapor R-3, Buchi, Switzerland). Afterward, the residue was redissolved in methanol (1 mL). The extract was filtered through a syringe filter (0.45 μm) for chromatographic analysis with UHPLC-DAD-MS*^n^*. Next, methanol was evaporated from the samples. The aliquots were dissolved in DMSO in proper concentrations for further investigation in the biological assays.

The control samples of fruit extract were incubated, with or without FS and BHI, according to the method previously described [23]. After 16 h incubation, the samples were collected. Healthy volunteers, aged 30–36, who did not suffer from any gastrointestinal disorders and had not used antibiotics for the previous six months, donated human fecal samples for in vitro experiments. Intake of tannin-, flavonoid-, and anthocyanin-containing products, including fruits and vegetables, coffee, and tea, was forbidden for 4 days before sample collection.

### 2.5. Inhibition of Pancreatic Lipase and α-Amylase by Digested Fractions

#### 2.5.1. Pancreatic Lipase Inhibitory Assay

The previously described method [24] was used to define the inhibitory potential of the extract on PL activity. As the enzyme source was used a porcine pancreas powder (10 mg/mL), which was dissolved in Tris-HCl buffer (pH 8.0). Lyophilized fractions were dissolved in DMSO solution. As a positive control, a standard inhibitor of PL such as orlistat was used. A sample without a fraction or a standard inhibitor was a control, which represented 100% activity of PL. The fluorescence of 4-methylumbelliferone was measured at λ_excitation_ = 360 nm and λ_emission_ = 465 nm at 37 °C in a microplate reader (Synergy 4, Bio Tek Instruments, Winooski, VT, USA). The concentration of the analyzed fractions ranged from 312.5 to 437.5 µg/mL.

#### 2.5.2. α-Amylase Inhibitory Assay

An EnzChek ultra amylase assay kit was used to analyze *α*-amylase activity based on the cleavage of a modified starch derivative, as previously described [24]. As the enzyme source was used a porcine pancreas powder (10 mg/mL), which was dissolved in Tris-HCl buffer (pH 8.0). Lyophilized fractions were dissolved in DMSO solution. As a positive control, a standard inhibitor of amylase acarbose was used. The sample without a fraction or a standard inhibitor was a control, which represented 100% activity of *α*-amylase. All the solutions—enzyme, substrate, and test sample—were prepared directly before assay. The fluorescence of the starch derivative was measured at λ_excitation_ = 485 nm and λ_emission_ = 535 nm at 37 °C in a microplate reader (Synergy 4, Bio Tek Instruments, Winooski, VT, USA). The concentration of the analyzed fractions ranged from 25 to 50 µg/mL.

### 2.6. HPLC Analysis of Digested Fractions

Chromatographic analysis was performed on a UHPLC-3000 RS system (Dionex, Germany) with a diode-array detector (DAD) and an AmaZon SL ion trap mass spectrometer with an ESI interface (Bruker Daltonik GmbH, Bremen, Germany). A Kinetex XB-C_18_ column (150 × 2.1 mm, 1.7 μm) (Phenomenex, Torrance, CA, USA) set at the temperature of 25 °C was used for separation. A mobile phase A was 0.1% formic acid in the water, and mobile phase B was 0.1% formic acid in acetonitrile. The gradient program with a flow rate 0.2 mL/min, used for the separation of phytochemicals, was as follows: 4–26% B, 0–60 min; 26–95% B, 60–90 min.; 4% (equilibration), 90–100 min. The UV chromatograms were registered at 240, 280, 325 nm, or 520 nm. The conditions of ESI parameters were described previously [22].

### 2.7. Kinetic Investigation of Extract Treated with Gut Microbiota

The crude extract of CM was treated with a suspension of human gut microbiota for 24 h. To evaluate the changes in its composition depending on the time, the phytochemical analysis was conducted after 1 h, 3 h, 6 h, and 24 h and referred to samples at time 0 h. The control samples of fruit extract at the concentration of 40 mg/mL were incubated, with or without FS and BHI, as it was previously described [23]. Healthy volunteers, aged 30–36, who did not suffer from any gastrointestinal disorders and have not used antibiotics for the last six months, donated human fecal samples for in vitro experiments. Intake of tannin-, flavonoid-, and anthocyanin-containing products, including fruits and vegetables, coffee, and tea, was forbidden for 4 days before sample collection.

The tested samples contained the CM extract solution at the concentration of 40 mg/mL (0.5 mL), 1 mL of FS suspension (1:10, *m*/*v*), and were filled up to 10 mL with BHI. The control samples contained 1 mL of FS suspension and 9 mL of BHI solution or 0.5 mL of extract and 9.5 mL of BHI. The suspensions were incubated in a container with GENbox anaer sachets at 37 °C. After each incubation, the 5 mL portions of samples were extracted with ethyl acetate (1:4, *v*/*v*). The residue was taken for further analysis with HPLC-DAD-MS*^n^* after evaporation of ethyl acetate under reduced pressure at 40 °C.

### 2.8. Statistical Analysis

Each sample was tested in triplicate in three independent experiments. The results are expressed as means ± SEM (standard error of the mean). Statistical significance of the differences between means was established by testing homogeneity of variance and normality of distribution followed by ANOVA and nonparametric methods such as the Mann–Whitney *U* test. The *P*-values below 0.05 were considered statistically significant. All analyses were performed using Statistica 13.3 (TIBCO Software Inc., Palo Alto, CA, USA) software.

## 3. Results

### 3.1. Inhibition of Digestive Enzymes by Gastrointestinal Fractions

**Figure 2:**
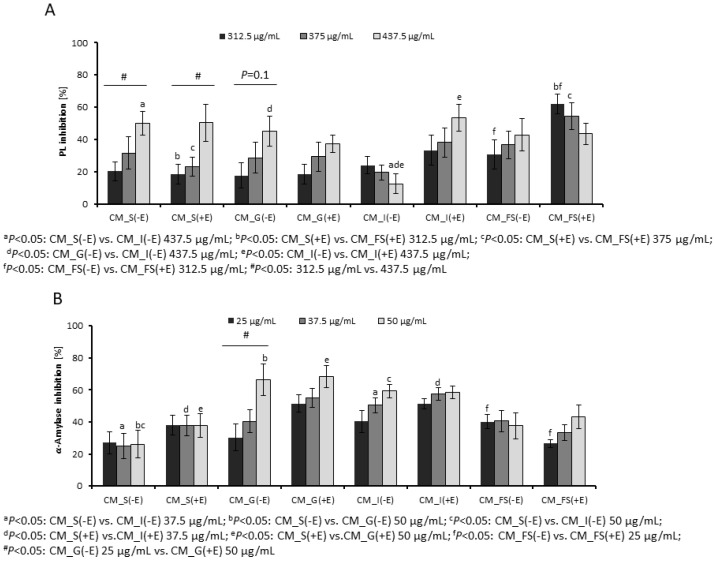
The inhibition of PL (**A**) and α-amylase (**B**) activities by fractions after gastrointestinal digestion of ethanolic-aqueous extracts from fruits of *C. mas* (CM) in vitro; CM_S-salivary fraction; CM_G-gastric fraction; CM_I-intestinal; CM_FS-fecal slurry/colon; (+E)-gastrointestinal pathway with digestive enzymes, (−E)-gastrointestinal pathway without digestive enzymes. The statistical analysis was performed with the Mann–Whitney *U* test. The pairs of letters indicate the statistical significance between samples.

The most active fractions of CM extract were CM_S(−E), CM_S(+E), CM_G(−E), CM_I(+E) at concentrations of 437.5 µg/mL inhibiting the PL activity by 50.02 ± 7.37%, 50.29 ± 11.46%, 45.06 ± 9.41%, and 53.55 ± 8.41%, respectively (Figure 2A). The most relevant differences between enzymatic and non-enzymatic pathways were observed in the CM_I fractions, as well as in the CM_FS fractions. The fraction CM_I(−E) (12.54 ± 6.27%) was a significantly less active inhibitor than CM_I(+E) (53.55 ± 8.41%) at concentration of 437.5 µg/mL. The fractions CM_FS(−E) and CM_FS(+E) inhibited PL activity by 30.67 ± 9.28% and 62.10 ± 6.11% at concentration of 312.5 µg/mL, respectively. A relevant inhibitory activity of FS control and BHI control after 16 h incubation allows to suspect a kind of interaction between constituents of CM, FS, and BHI, leading to alteration of their inhibitory potential [22]. The IC_50_ value for the standard PL inhibitor, orlistat, was 11.6 ng/mL.

Among CM fractions, the CM_G and CM_I ones were the most active inhibitors of *α*-amylase activity (Figure 2B). The inhibition of *α*-amylase by CM_G(−E) and CM_G(−E) at a concentration of 50 µg/mL reached even the values of 66.22 ± 9.91% and 68.30 ± 6.92%, respectively. The *α*-amylase inhibition caused by CM_I fractions also exceeded 50%. The metabolic transformation of CM fruit extract with FS reduced this activity to 43.23 ± 7.53% for CM_(+FS). The positive control, acarbose, at the concentration of 2.4 µg/mL lowered the activity of *α*-amylase by 50%.

### 3.2. The Phytochemical Analysis of Gastrointestinal Fractions

**Figure 3:**
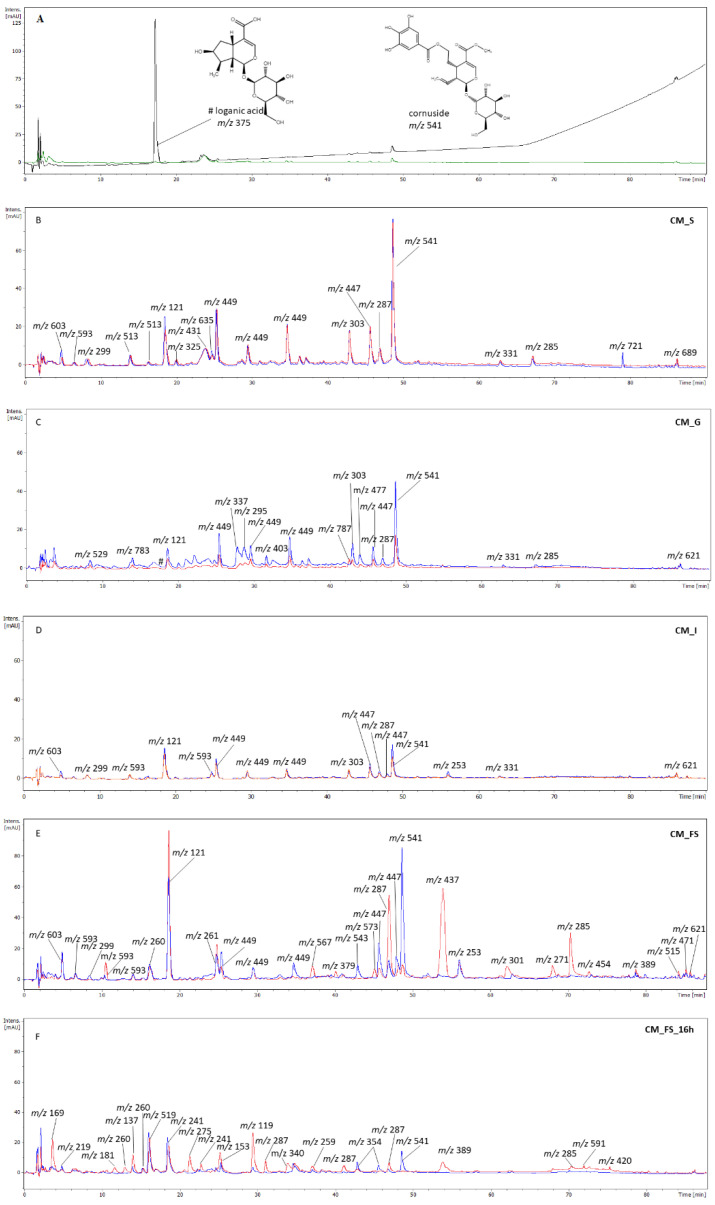
UV (λ = 280 nm) chromatograms of fractions after gastrointestinal digestion of ethanolic-aqueous extracts from fruits of *C. mas* in vitro. CM–crude extract of *C. mas* fruits (**A**); CM_S—salivary fraction (**B**); CM_G—gastric fraction (**C**); CM_I—intestinal (**D**); CM_FS—fecal slurry/colon (**E**); CM_FS_16 h—control CM extract incubated with FS for 16 h (**F**). Red line—gastrointestinal pathway with digestive enzymes or FS; blue line—gastrointestinal pathway without neither digestive enzymes nor FS. ^#^ *m*/*z* 783 in the negative ESI mode.

The major compound in the CM extract was loganic acid ([M − H]^−^ *m*/*z* 375) from the class of iridoids, which was registered at R_t_ = 17.7 min (Figure 3). It seems that thanks to the presence of carboxylic moiety, it easily transforms to an adduct form described as the main ion in the MS spectrum were [2M − H]^−^ (*m*/*z* 751) in negative ESI mode [25]. Loganic acid absorbs UV at λ = 240 nm, but it could not have been detected at λ = 280 nm, which is, on the other hand, more suitable for investigation of other iridoids or compounds such as phenolic acids (Figure 3A). The peak of loganic acid was not detected in the salivary fraction just after 5 min of incubation. However, it appeared on the chromatogram of gastric fraction (R_t_ = 17.7 min) at λ = 240 nm (Figure 3C). It was completely digested in the intestinal (Figure 3D) and colon (incubation with FS) (Figure 3E) fractions. Another iridoid found in CM extract is cornuside, of which the main ion [M − H]^−^ *m*/*z* 541 was detected in the negative ESI mode at R_t_ = 48.9 min [25]. It was found in most analytes such as salivary, gastric, intestinal, and even CM_FS(+E) fractions. It was shown that gut microbiota suspension in CM_FS(+E) significantly affects the digestion of cornuside compared with CM_FS(−E) fraction (blue line, Figure 3E). It is worth noting that the peak of cornuside in CM_FS_16 h was completely lost, but still found in CM_BHI_16 h (blue line, Figure 3F). Therefore, the presence of cornuside in the FS fraction derived from the gastrointestinal pathway and its loss in the fraction when the crude CM extract was digested directly by FS, despite the same time of incubation of fractions, allows suspecting that the matrix of phytochemicals from gastrointestinal digestion protects cornuside from complete digestion.

The major anthocyanin was pelargonidin-3-*O*-galactoside (R_t_ = 24.0 min). The main ion in its MS spectrum was [M + H]^+^ (*m*/*z* 433), whereas its major MS^2^ fragmentation pattern showed a signal at *m*/*z* 271 in positive ESI mode [25]. Apart from the crude extract, as well as salivary and gastric fractions, this compound was not detected in analytes [CM_I(−E), CM_I(+E), CM_FS(+E)] from further imitated gastrointestinal compartments, except for CM_FS(−E). Moreover, a signal at *m*/*z* 449 [M − H]^−^ and *m*/*z* 451 [M+H]^+^ in the negative and positive ESI mode, respectively, was registered at R_t_ = 34.9 min. Taking into consideration the MS^2^ fragmentary pattern such as *m*/*z* 269 [M − H]^−^ and *m*/*z* 271 [M+H]^+^, we suppose that it may be a pelargonidin derivative such as pelargonidin hexuronide.

Among flavonoids, the derivatives of kaempferol were mostly detected. In all fractions, compounds, which provided the main ion *m*/*z* 447 [M − H]^−^ in negative ESI mode, were detected and tentatively assigned to kaempferol hexosides (R_t_ = 45.7 min and R_t_ = 46.9 min) [26]. Its major MS^2^ fragmentation pattern showed a signal at *m*/*z* 285. The aglycone of kaempferol was registered at R_t_ = 67.1 min in salivary and gastric fractions. In addition, its isomer was registered at R_t_ = 70.5 min. Furthermore, in all gastrointestinal fractions, aromadendrin was detected and assigned based on the main ion *m*/*z* 287 [M − H]^−^ at R_t_ = 47.2 min [27]. It is supposed that in the limited air conditions, the aglycone of kaempferol is reduced to aromadendrin. It is worth noting that the peak of aromadendrin was even more intensive in FS(+E) fraction than in FS(−E), which probably means that enzymatic hydrolysis of aromadendrin hexoside takes place in the bacterial environment. The main ion of aromadendrin hexosides was *m*/*z* 449 [M − H]^−^ in the negative ESI mode registered at R_t_ = 25.6 min and R_t_ = 29.7 min. They were found in all fractions of the gastrointestinal pathway.

Another flavonol identified in the gastrointestinal fractions after digestion of CM extract was quercetin and its derivatives. In the gastric fractions, a compound characterized by the main ion *m*/*z* 477 [M – H]^−^ was noted at R_t_ = 45.1 min. Its fragmentary ion was *m*/*z* 301 in negative ESI mode. Therefore, the compound was tentatively identified as quercetin glucuronide [26,28]. In addition, in the salivary, gastric, and intestinal fractions dihydroquercetin (syn. Taxifolin) was assigned. In negative ESI mode, its main ion was *m*/*z* 303 [M − H]^−^ at R_t_ = 43.1 min, whereas the fragmentary ion was *m*/*z* 163 [29].

In all gastrointestinal fractions obtained in vitro, the signals corresponding to tannins were detected. The compounds, of which major ions in the MS spectrum were *m*/*z* 513 [M − H]^−^ (R_t_ = 14.0 min and R_t_ = 16.4 min), were found in the negative ESI mode (Figure 3B). Their MS^2^ fragmentation pattern showed signals at *m*/*z* 495, 343, 271, 241, and 169. The compounds were assigned to gallic acid derivatives. The isomers of the compound, which showed a signal at *m*/*z* 593 (R_t_ = 6.5 min and R_t_ = 16.4 min) in the negative mode (CM_S, CM_I, and CM_FS), provided fragmentary ions such as 547, 513, 503, and 333. Similar fragmentary ions were found in the case of a compound described by *m*/*z* 603 in the negative ESI mode. In some analytes, the MS^2^ fragmentation pattern of the ion showed a signal at *m*/*z* 337 in the negative ESI mode. Therefore, we suppose that it might be a derivative of *p*-coumaroylquinic acid.

The compounds identified as hydrolyzable tannins were found at R_t_ = 14.0 min and R_t_ = 18.6 min (CM_G). The major ion in their MS spectrum was *m*/*z* 783 [M − H]^−^. Its major MS^2^ fragmentation pattern showed a signal at *m*/*z* 483 characteristic for digalloyl glucose. Therefore, they were tentatively assigned to cornusiin A [30]. The ion providing a signal at *m*/*z* 787 (FS_(−E) (R_t_ = 42.5 min), as well as fragmentary ions at *m*/*z* 635 (tri-*O*-galloyl-*β*-D-glucose), 617, 465, and 303, was tentatively assigned to tetra-*O*-galloyl-*β*-D-glucose in CM_G [30].

Benzoic acid, of which the main ion is [M − H]^−^ *m*/*z* 121, was detected in the negative ESI mode at R_t_ = 18.7 min in analytes from salivary to FS fractions. Its amount seems to increase significantly in particular in the analytes incubated with FS. In addition, an ion [M − H]^−^ *m*/*z* 299, found in all gastrointestinal fractions except for gastric one, was tentatively assigned to hexoside of hydroxybenzoic acid [M − H]^−^ *m*/*z* 137.

### 3.3. The Analysis of Time-Dependent Changes of Metabolites after Gut Microbiota Treatment

**Figure 4:**
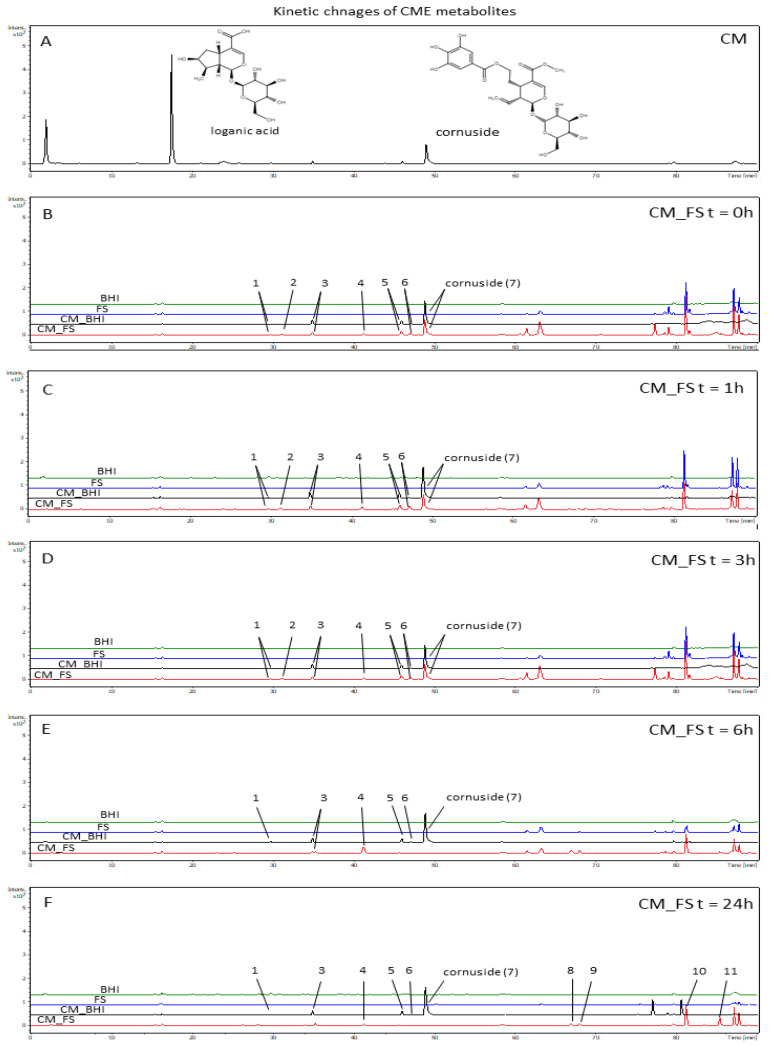
Ion chromatograms of ethanolic-aqueous extract from fruits of *C. mas* (CM; (**A**)) after the treatment with FS for 0 h (**B**), 1 h (**C**), 3 h (**D**), 6 h (**E**), and 24 h (**F**).

The kinetic changes of metabolites of CM extract were registered after the treatment of crude extract with FS for 0 h, 1 h, 3 h, 6 h, and 24 h. Total ion chromatograms in negative ESI mode were presented in Figure 4, whereas UV chromatograms were attached as Supplementary Materials (Figures S1–S5). In general, the metabolites originating from kinetic analytes have been classified mainly as phenolic acids, hydrolyzable tannins, and flavonoids. In Table 2, the data on the main and fragmentary ions are provided for the major compounds. The most abundant signals in the analytes from 0 h to 24 h were derived from aromadendrin derivatives. The signals tentatively assigned to pelargonidin hexuronide, kaempferol hexoside, and cornuside were digested successively (Table 2).

The analysis of transformation of CM metabolites after ex vivo FS digestion for 24 h showed mostly phenolic derivatives such as gallic acid [*m*/*z* 169 [M − H]^−^ (R_t_ = 3.7 min)], hydroxybenzoic acid [*m*/*z* 137 [M − H]^−^ (R_t_ = 14.1 min)], and protocatechuic acid [*m*/*z* 153 [M − H]^−^ (Rt = 25.6 min)]. Protocatechuic acid was noted in further analytes after 6 and 24 h of incubation with FS. It is supposed that protocatechuic acid might originate from the transformation of anthocyanins [31]. Despite the peak of hydroxybenzoic acid found in the analytes treated with FS in t = 0 h, 1 h, and 3 h, it was tentatively identified based on MS spectrum in the samples obtained after 6 h and 24 h incubation of CM with FS. After 6 and 24 h, a signal at *m*/*z* 165 [[M − H]^−^ (R_t_ = 30.2 min) appeared. It was tentatively assigned to a carboxylic derivative of benzoic acid. In the meantime, the peak assigned to aromadendrin hexoside was completely digested after 6 and 24 h.

The peak of a compound at *m*/*z* 635 [M − H]^−^ (R_t_ = 24.4 min) was identified as tri-*O*-galloyl-*β*-D-glucose. The loss of 152 amu, the fragmentary signals at *m*/*z* 483 [M − H]^−^ and *m*/*z* 301 [M − H]^−^ allowed us to suspect that it was a gallotannin providing a significant signal in CM_FS_6h. In the FS-treated analytes, the ions such as [M − H]^−^ *m*/*z* 483 (R_t_ = 8.0 min and R_t_ = 4.3 min) were found and tentatively assigned to diglalloylglucose. The MS^2^ fragmentation pattern of [M − H]^−^ *m*/*z* 483 was 331 and 169. After 3 h incubation of extracts with FS, the peak of gallic acid at *m*/*z* 169 [M − H]^−^ (R_t_ = 3.7 min) was also detected. It is concluded that gallic acid derives from the hydrolysis of gallotannins. On the other hand, it might easily react with sugars-derived products of hydrolysis. Gallotannins may be formed in this manner in the analyzed samples. On the other hand, in the further CM_FS analytes, few signals from unidentified compounds were detected *m*/*z* 887 (R_t_ = 58.4 min), *m*/*z* 745 (R_t_ = 67.0 min), *m*/*z* 595 (R_t_ = 68.1 min), *m*/*z* 391 (R_t_ = 81.2 min), and *m*/*z* 297 (R_t_ = 85.4 min) (Table 2).

The main ion of kaempferol hexoside *m*/*z* 447 [M − H]^−^ was registered in the analytes before and after 1 h as well as 3 h incubation with FS. Aromadendrin *m*/*z* 287 [M − H]^−^ was detected at R_t_ = 47.0 min in all analytes after incubation with FS. Few isomers were detected (Table 2). Dihydroquercetin [M − H]^−^ *m*/*z* 303 (R_t_ = 43.1 min) was identified in the FS analytes after 6 h of incubation. The MS^2^ ion of dihydroquercetin pattern was *m*/*z* 165.

It is worth noting that an iridoid compound such as cornuside was found in FS analyte after 3 h of incubation, which proves its resistance to enzymatic digestion and makes it a potentially bio-accessible constituent of CM extract. The compound was stable even after 24 h incubation with BHI.

## 4. Discussion

Obesity is considered a disorder associated with a state of chronic low-grade inflammation, which is developed in response to excessive application of nutrients. It is worth noting that some of the plant materials and their phytochemicals are characterized by suppressing appetite/hunger and/or increasing satiety properties [32]. On the other hand, the phytochemicals in particular found in nutritional plant materials demonstrate indirect antioxidation-related or direct anti-inflammatory activity [33]. The identification of compounds potentially responsible for this effect seems to be necessary. Taking into consideration that extracts from cornelian cherries are considered anti-diabetic and anti-obesity preparations, we performed the preliminary research evaluating the changes of secondary metabolites of the CM extract and the effect of its postprocessing fractions on the activity of enzymes, key for the absorption of lipids and sugars. According to our knowledge, there is no study concerning changing of secondary metabolites found in cornelian cherries.

Obesity and hyperlipidemia are linked with risk factors such as insulin resistance, impaired glucose tolerance, the severity of liver fibrosis, and hypertension. Therefore, prevention and treatment of obesity are crucial for the reduction of the prevalence and mortality due to chronic metabolic diseases. Among lipases in the digestive system, including the tongue, gastric, and pancreatic lipases, pancreatic lipase is the primary lipase secreted from the pancreas. It is directly responsible for the intestinal absorption of fatty acids. It hydrolyzes dietary lipids derived from oil or fat, converting triacylglycerol substrates to free fatty acids and monoglycerides. It is responsible for 50–70% of decomposition, whereas tongue lipase digests lipids to a small extent and gastric lipase decomposes 10–30% of fat. In the intestine, free fatty acids and monoglycerides are moved to enterocytes, where they are absorbed and take a part in metabolism by the formation of cholesterol and lipoprotein. Inhibitors of PL play a key role in the metabolism of dietary fat. Two categories of medicines, including inhibitors of intestinal absorption of fat (orlistat) and suppressant of appetite acting on the central nervous system (fenfluramine, sibutramine), are currently used for weight loss. In particular, appetite suppressants are known for their serious side effects on the central nervous system. Lipase inhibitors are considered relatively safe. They provide some advantages in acting on peripheral elements in the gastrointestinal tract but not entering human blood vessels or the nervous system. In addition, they do not affect the balance of bone circulation and the body’s minerals. It seems that the disadvantages of orlistat, such as oily stools, are less dangerous for patients [34].

On the other hand, amylase is a calcium metalloenzyme produced by salivary glands. It catalyzes *α*-(1,4)-D-glycosidic linkages present in starch molecules providing breakdown products such as maltose, which in turn cleaves into two molecules of glucose. In further parts of the gastrointestinal tract, the digestion of starch is continued by pancreatic amylase released through the pancreatic duct to the duodenum. The inhibiting *α*-amylase enzyme helps in reducing hyperglycemia, obesity, and problems linked with overweight conditions. Various enzymatic inhibitors, such as acarbose, miglitol, and voglibose, are effective in targeting this enzyme. Usage of inhibitors of *α*-amylase is one of the ways to lower postprandial hyperglycemia by reducing the hydrolysis rate of dietary starch. Among plant-derived products, in particular, flavonoids are indicated as inhibitors of this enzyme [35].

We established that gastric and intestinal fractions at a concentration of 50 µg/mL were particularly active inhibitors of *α*-amylase. In both of them, the compounds belonging to a group of polyphenols, such as flavonoids, phenolic acids, anthocyanins, and traces of tannins, were detected. We focused our attention on the flavonols, such as aromadendrin (*m*/*z* 287), kaempferol (*m*/*z* 285), and dihydroquercetin (*m*/*z* 303), which appeared in many fractions. Bearing in mind that cornuside and anthocyanins were the most abundant compounds in salivary fractions, which were less active than gastric and intestinal ones, it is supposed that they do not significantly influence the activity of tested enzymes. Indeed, in our previous study, we established that cornuside isolated from cornelian cherries did not inhibit the PL activity. In that study, we also concluded that flavonoids are more relevant inhibitors of digestive enzymes than anthocyanins and iridoids. In addition, in the gastric fractions, we assigned the compounds from the class of tannins. It was previously established that IC_50_ values for kaempferol-3-*O*-glucoside, protocatechuic acid, gallic acid, and catechin were as follows—5.57 ± 0.46 mg/mL, 1.78 ± 0.07 mg/mL, 1.27 ± 0.04 mg/mL, and 2.44 ± 0.11 mg/mL, respectively, in the case of *α*-amylase activity. The same compounds inhibited PL activity, showing the following IC_50_ values: 1.58 ± 0.13 mg/mL, 1.03 ± 0.09 mg/mL, 0.86 ± 0.041 mg/mL, and 0.53 ± 0.05 mg/mL [36]. It might be concluded that the complex composition of the fractions containing a wide range of phenolics might exert a more relevant effect when compared with pure compounds. That allows us to suspect that phenolic compounds can inhibit amylase and lipase in the gastrointestinal tract even at low doses. On the other hand, a system of digestive enzymes, including carbohydrate-hydrolyzing enzymes, lipases, and proteases, is involved in the digestive process in the gastrointestinal tract. In our model of in vitro gastrointestinal digestion of CM, we used digestive enzymes such as salivary amylase, pepsin, and pancreatin. To provide any control, we used two pathways of digestion, including or not digestive enzymes occurring in the gastrointestinal tract. The nonrelevant changes were observed between these pathways, particularly in compartments from salivary to intestinal, where the artificial enzymes were used. We realize the limitation of this method due to the possible inhibition of digestive enzymes used in the assay by metabolites of cornelian cherries. In particular, the inhibition of digestive enzymes by phenolic compounds from herbal and fruit extracts was widely revised [37]. This type of inhibition prevents the potential hydrolysis of extract constituents. For this reason, the enzymatic digestion of phytochemicals might be limited and not provide sufficient data on phytochemical changes during digestion products. Therefore, the significant differences between the activities of fractions derived from pathways with or without enzymes were probably not observed, except for CM_I at a concentration of 437.5 µg/mL in PL assay. Gastrointestinal slurries ex vivo seem to be more suitable and preferable to assess the metabolism of phytochemicals. However, it must be underlined that the acquisition of gastrointestinal slurry is limited. The stool is relatively easy for sampling. For this reason, it is often used as a proxy for the intestinal microbiota as it was in the case of the present study. Apart from physicochemical conditions, the intestinal compartments are characterized by different microbial populations. In the small intestine, bacteria such as *Lactobacillaceae*, *Bacteroidales*, and *Desulfovibrionaceae* occur in addition to simple nutrients. Other species from *Proteobacteria* (mainly *E. coli*) and *Streptococcus* spp. colonize the ileum. On the other hand, the fecal slurries from the large intestine are enriched in anaerobes belonging to Firmicutes and Bacteroidetes, such as families of *Bacteroidaceae, Lachnospiraceae*, *Prevotellaceae*, *Ruminococcaceae*, and *Rikenellaceae* [38]. There is no doubt that thanks to the microorganisms, the fate of phytochemicals differs in the small and large intestine. In the small intestine, metabolized nutrients are absorbed by the host or depleted by microbiota, whereas in the large intestine, anaerobes ferment polysaccharides and other dietary compounds. However, bacteria-derived hydrolyzing enzymes from both intestinal and colonic compartments may participate in the metabolism of phytochemicals. In our study, the fecal slurry was used to mimic the bacterial role in the digestion of phytochemicals, which takes place in the different compartments of the gastrointestinal tract in vivo. We suppose that metabolites of cornelian cherries similar to that formed in the FS compartment of our study may occur in the human intestine and inhibit the hydrolysis of fats and sugars via the inhibition of hydrolyzing enzymes. To the best of our knowledge, there are no data on the metabolites of cornelian cherries. Nevertheless, further studies including slurries from upper parts of the gastrointestinal tract from animal models as well as isolation and more detailed identification of metabolites should be developed.

It was previously widely reported that loganic acid is the most abundant compound in CM preparations [25]. Our study clearly shows that loganic acid is unstable in basic and neutral pH conditions. Another iridoid, cornuside, belongs to the specific class of secoiridoids. Its antioxidant and anti-inflammatory activities have been proven [17,39,40]. Additionally, it is also likely to have beneficial effects on Alzheimer’s disease and myocardial ischemia, and reperfusion injuries [41,42]. The presence of cornuside in the colon (FS)-derived fraction from gastrointestinal digestion in vitro makes it potentially bio-accessible. In addition, cornuside was even found in analytes after 6 h digestion with porcine intestinal slurries ex vivo (data not shown), but these data require more detailed investigation. Therefore, previous reports on the activity of isolated cornuside are justified and their results might be related to in vivo effects.

On the other hand, the effect of cornuside on bacterial growth might be worthy of further investigation. It has been already proven that it alleviates diabetes mellitus through modulation of gut microbiota. Cornuside reversed the changes in the distribution of gut microbiota and decrease the abundance of *Clostridium* sp. ND2, *Weissella confusa*, *Anaerotruncus colihominis* DSM 17241, *Roseburia*, [*Clostridium*] *leptum* (*Anaerotruncus*), *Ruminococcus*, and *Bilophila* in a fecal slurry of KK-Ay mice [43].

Apart from iridoids, metabolites of anthocyanins and flavonoids are worth specific consideration. The class of flavonoids, which are represented by aglycones, such as aromadendrin, kaempferol, quercetin, and dihydroquercetin, in the digested fractions in our study, is supposed to affect the gut microbiota. They inhibit the generation of reactive oxygen species, which lead to oxidative stress and, in consequence, inflammation [44]. Their antioxidant activity may additionally protect the intestinal epithelium. It is believed that homeostasis of gut microbiota is pivotal for assuring health benefits. In particular, the antibacterial properties of flavonoids allow for inhibiting the growth of pathogenic bacteria [45]. On the other hand, their probiotic activity supporting the growth of strains such as *Bifidobacterium* and *Lactobacillus* should be taken into consideration. In general, polyphenolic compounds influence the activity of enzymes engaged in the metabolism of carbohydrates and lipids, as well as the secretion of intestinal hormones [46]. Among flavonol aglycones, dihydroquercetin was detected after the treatment with porcine FS in our preliminary studies (data not shown). Additionally, quercetin glucuronide was found in our gastric analytes. It is known that glucuronides are usually formed as the end-product of the I and II phases of metabolism [28]. We suppose that acidic conditions promote the formation of uronic acids from sugars derived from acid hydrolysis of glycosides. It was established that cornelian cherries contain 255.4 to 457.2 mg polyphenols, including flavonoids, phenolic acids, and tannins, expressed as gallic acid/100 g fresh weight [47]. Therefore, it is hypothesized that the supplementation of polyphenol-rich extract of cornelian cherries may be a way to achieve homeostasis of gut microbiota. According to the available literature, dysbiosis of gut microbiota is considered a factor in the progression of insulin resistance in type 2 diabetes. In addition, it may reshape intestinal barrier functions, as well as further host metabolic and signaling and metabolic pathways. Thus, it is directly or indirectly related to insulin resistance [48]. Taking into consideration the antidiabetic prerequisites of cornelian cherries, this is the proposed mode of action for this plant material. However, further investigation is necessary to support this hypothesis. Direct studies of gut microbiota upon treatment with fruits of *C. mas* will allow us to conclude.

In particular, the derivatives of aromadendrin, including aromadendrin itself, were often registered in all studied analytes, both intestine- and colon-derived. It is believed that enzymes from bacteria, as well as changing pH in the distal parts of the gastrointestinal tract, significantly participate in the metabolism of glycosides [49,50]. The enzymatic hydrolysis is believed to take place in the small intestine where some bacteria, such as *Proteobacteria*, *Streptococcus* spp., and *Lactobacillaceae*, are found [38].

Our qualitative analysis of metabolites from CM extract shows the presence of phenolic compounds in digested fractions. We tentatively assigned them to gallic acid, protocatechuic acid, and benzoic or hydroxybenzoic acid. It is worth noting that protocatechuic acid was reported as a product of anthocyanin degradation [51]. Phenolic acids, such as *p*-coumaroylquinic acids [M − H]^−^ *m*/*z* 337, were previously identified in extract of *C. mas* [26]. On the other hand, hexahydroxydiphenolic acid [M − H]^−^ *m*/*z* 337 linked with polysaccharides or phenolic acids through ester bonds forms ellagitannins. The signal at *m*/*z* 337 might be a result of ellagitannins hydrolysis. The released hexahydroxydiphenolic acid is converted to ellagic acid by lactonization [52]. Nevertheless, we identified no ellagitannins, contrary to findings on gallotannins. We suppose that they derive rather from the stones of fruits, as it was recently published [30], or they are formed throughout digestion. Gallic acid is the final product of their digestion. Phenolic compounds, including phenolic acids and flavonoids, are particularly active inhibitors of PL and *α*-amylase [36,53]. The gastric and intestinal fractions of digested CM extract particularly inhibited *α*-amylase activity in the concentrations up to 50 µg/mL. On the other hand, the activity of lipase was inhibited by 50% in the case of fractions in the highest tested concentrations (437.5 µg/mL). That may support the lipid lowering prerequisites, in particular triglicerides, in the previous studies in vivo [11].

In general, the long-term storage and temperature increase may lead to the reduction of polyphenolic content in the preparations of cornelian cherries. On the other hand, it was established that flavonoids, rather than anthocyanins, are stable in the tinctures [47]. It was previously reported that the supplementation of alcoholic preparation from apples with juice from cornelian cherries significantly improved the antioxidant properties of the main product or even prolonged the expiry date of food products [54,55]. Furthermore, supplementation of beef with juice from cornelian cherries caused a decrease in lipid oxidation. Thus, the antioxidant compounds of CM extracts protect meat, assuring their quality as well as taste attributes in addition to health benefits for consumers [56]. In the study by Czyżowska et al. (2018), the fermentation of unripe cornelian cherries with lactic acid resulted in a product proposed as the source of probiotic bacteria for preventing dysbiosis of gut microbiota or as an alternative for patients suffering from malfunctioning lactose digestion [57]. In our study, the ethanolic-aqueous extract of CM was treated with gastrointestinal and bacteria-derived enzymes to check the stability of CM phytochemicals in such conditions, as well as to provide the data on potential postbiotic metabolites of cornelian cherries, which may support the functionality of gut microbiota.

The present study shows for the first time the possible pathways of metabolites formation when CM extract is digested. We hypothesized that cornuside, aromadendrin, and phenolic acids should be particularly considered as the compounds altering or alleviating obesity-related diabetes mellitus or other metabolic disorders.

## 5. Conclusions

The results of the present research allowed us to assess the stability of the most abundant compounds of cornelian cherries depending on the pH and enzymes of the gastrointestinal tract. The digestion of the phytochemicals with bacterial enzymes found in the upper parts of the gastrointestinal tract in vivo might also influence the metabolism of these compounds. Nevertheless, this suspicion requires further investigation in vivo. However, this is the first study considering the digestive changes of preparations from cornelian cherries according to our knowledge. The most abundant constituent of the studied extract, loganic acid, was completely digested. On the other hand, cornuside, the second best-known iridoid of *C. mas* turned out to reach the intestinal compartment and be partially resistant to bacterial enzymes from the human fecal slurry. Therefore, its potential bio-accessibility should be considered. Moreover, all metabolites found in the intestinal or colon compartments, such as phenolic acids, flavonoids, and gallotannins, should be taken into account as far as their anti-amylase and anti-lipase activities are concerned. Thus, it may partially explain the use of cornelian cherries in diabetes and obesity complaints. However, further identification of metabolites, including the isolation and confirmation of structures with spectroscopic methods, as well as in vivo studies concerning the pharmacokinetics of potentially active phytochemicals, are still necessary.

## Figures and Tables

**Table 1:** Composition of gastrointestinal fractions in the imitated digestion model in vitro.

Medium	Salivary Fraction	Gastric Fraction	Intestinal Fraction	Colon Fraction
Enzymes	α-amylase (1 U/μL)	pepsin (7.4 mg/mL)	pancreatin (2 mg/mL)	fecal slurry (1:10, *m*/*v*)
pH	6.8	2.0	6.9	-
Additives	KCl (110 g/L)			
	KSCN (25 g/L)			
	NaH_2_PO_4_ (110 g/L)		bile salts (25 mg/mL)	
	Na_2_SO_4_ (70 g/L)	HCl (150 mM)	NaHCO_3_ (0.5 M)	BHI
	NaCl (220 g/L)			
	NaHCO_3_ (105 g/L)			
	urea (30 g/L)			

**Table 2:** MS spectrum data of compounds detected in fractions incubated with FS depending on time.

No.	Retention Time [min]	λ_max_ [nm]	[M − H]^−^	MS2^−^	Assigned Compound
1	29.7	350	449	431, 355, 329, 287, 269	aromadendrin hexoside
2	31.2	265, 310, 364	287	259	aromadendrin isomer
3	34.9	260, 300, 350	449	431, 287, 269	pelargonidin hexuronide
4	41.3	265, 310, 364	287	269, 243, 165	aromadendrin isomer
5	45.7	345	447	327, 284	kaempferol hexoside
6	47.2	270, 310, 365	433	287	aromadendrin
7	49.0	280	541	379	cornuside
8	67.0	265, 310, 350	745	630, 388. 257	unidentified
9	68.1	270, 320, 360	595	549, 505, 462	unidentified
10	81.2	270, 320, 355	391	345	unidentified
11	85.4	260, 310, 350	297	279, 185	unidentified

## Data Availability

Not applicable.

## Supplementary Materials

The following supporting information can be downloaded at: https://www.mdpi.com/article/10.3390/nu14112287/s1. Figure S1. HPLC chromatograms of ethanolic-aqueous extract from fruits of *C. mas* treated with FS in t = 0 h registered at 240, 280, and 325 nm; Figure S2. HPLC chromatograms of ethanolic-aqueous extract from fruits of *C. mas* treated with FS in t = 1 h registered at 240, 280, and 325 nm; Figure S3. HPLC chromatograms of ethanolic-aqueous extract from fruits of C. mas treated with FS in t = 3 h registered at 240, 280, and 325 nm; Figure S4. HPLC chromatograms of ethanolic-aqueous extract from fruits of C. mas treated with FS in t = 6 h registered at 240, 280, and 325 nm; Figure S5. HPLC chromatograms of ethanolic-aqueous extract from fruits of C. mas treated with FS in t = 24 h registered at 240, 280, and 325 nm.
