# Supplementary material for: Potentially Bio-Accessible Metabolites from an Extract of Cornus mas Fruit after Gastrointestinal Digestion In Vitro and Gut Microbiota Ex Vivo Treatment
Source: Nutrients. 2022 May 30;14(11):2287. doi: 10.3390/nu14112287 (PMC9183047; doi:10.3390/nu14112287)
Supplement: Supplementary file 1 [file nutrients-14-02287-s001.zip › nutrients-1726324-supplementary.pdf]

nutrients

MDPI

Supplementary Materials

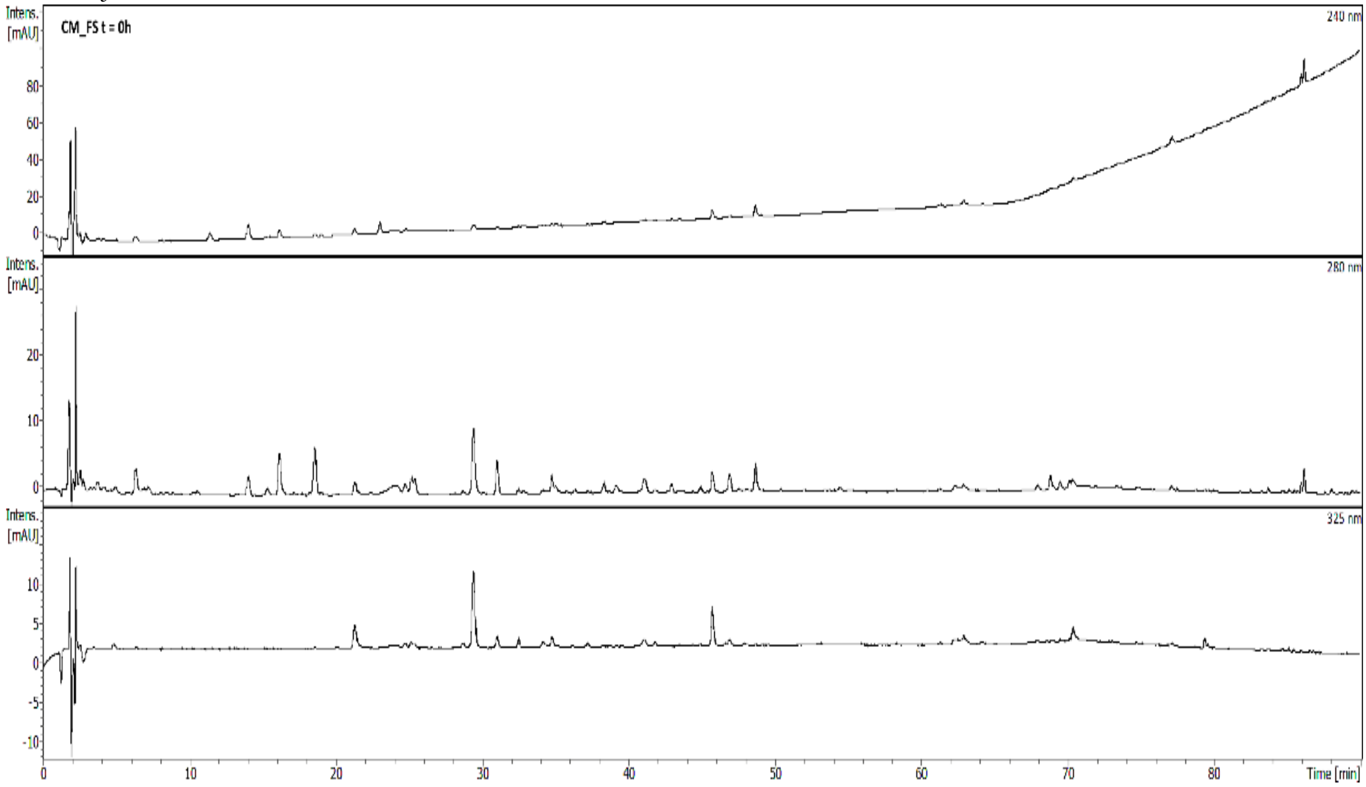

Figure S1. HPLC chromatograms of ethanolic-aqueous extract from fruits of *C. mas* treated with FS in t = 0 h registered at 240, 280, and 325 nm.

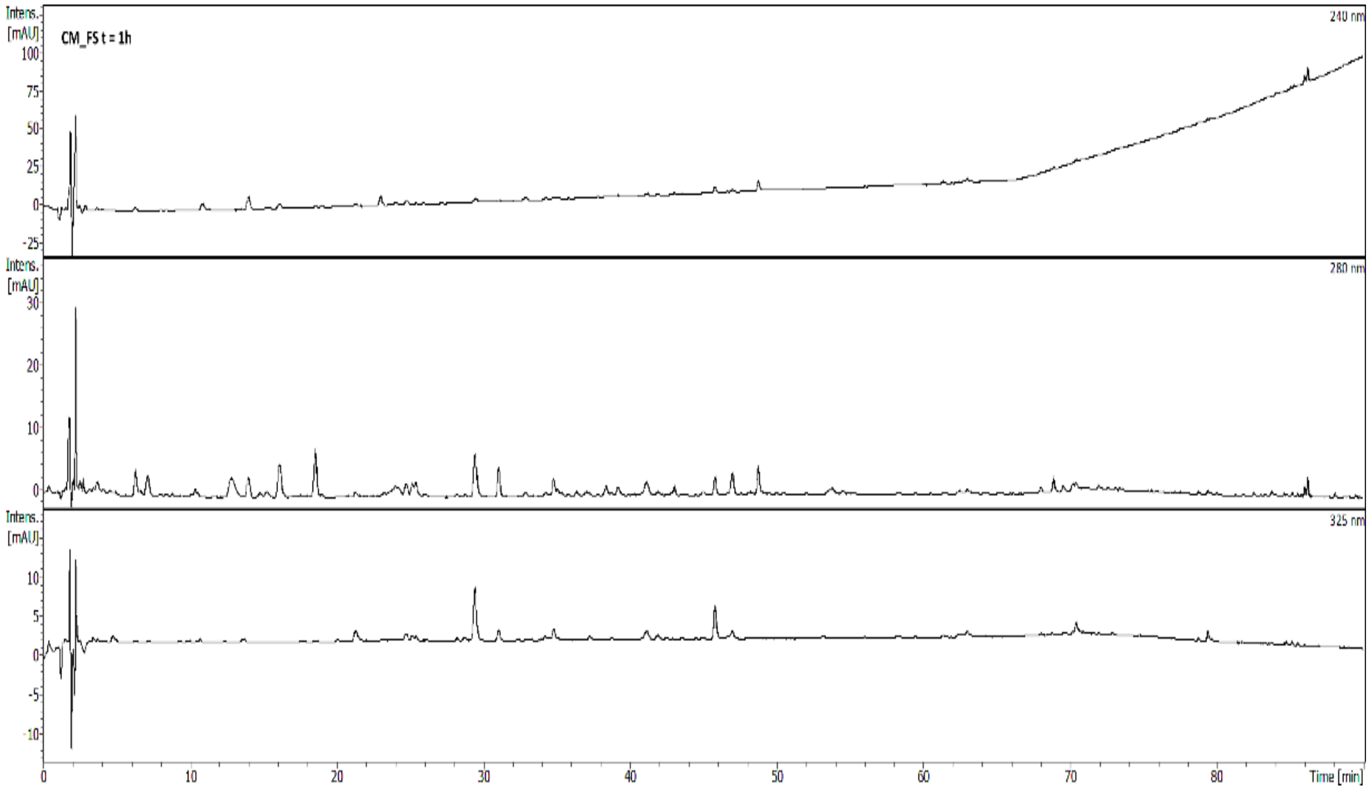

Figure S2. HPLC chromatograms of ethanolic-aqueous extract from fruits of *C. mas* treated with FS in t = 1 h registered at 240, 280, and 325 nm.

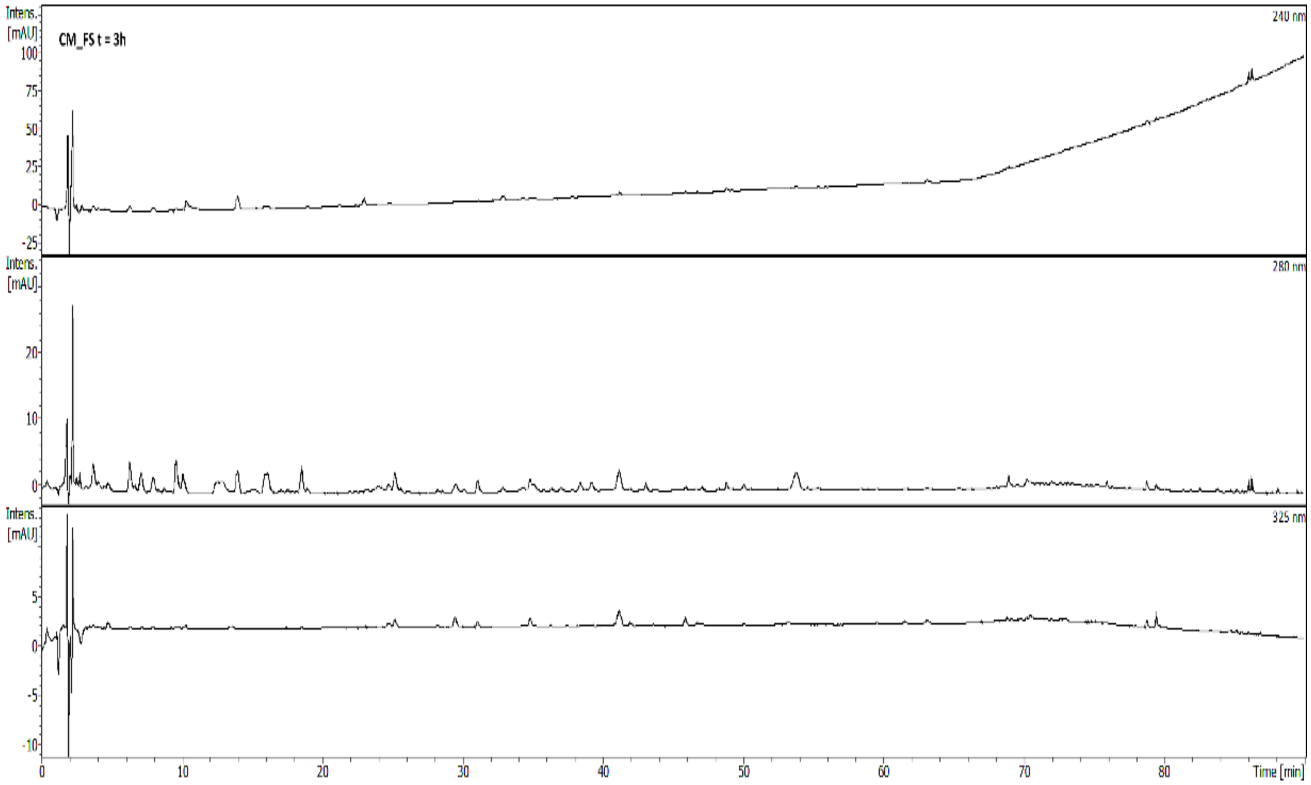

Figure S3. HPLC chromatograms of ethanolic-aqueous extract from fruits of *C. mas* treated with FS in t = 3 h registered at 240, 280, and 325 nm.

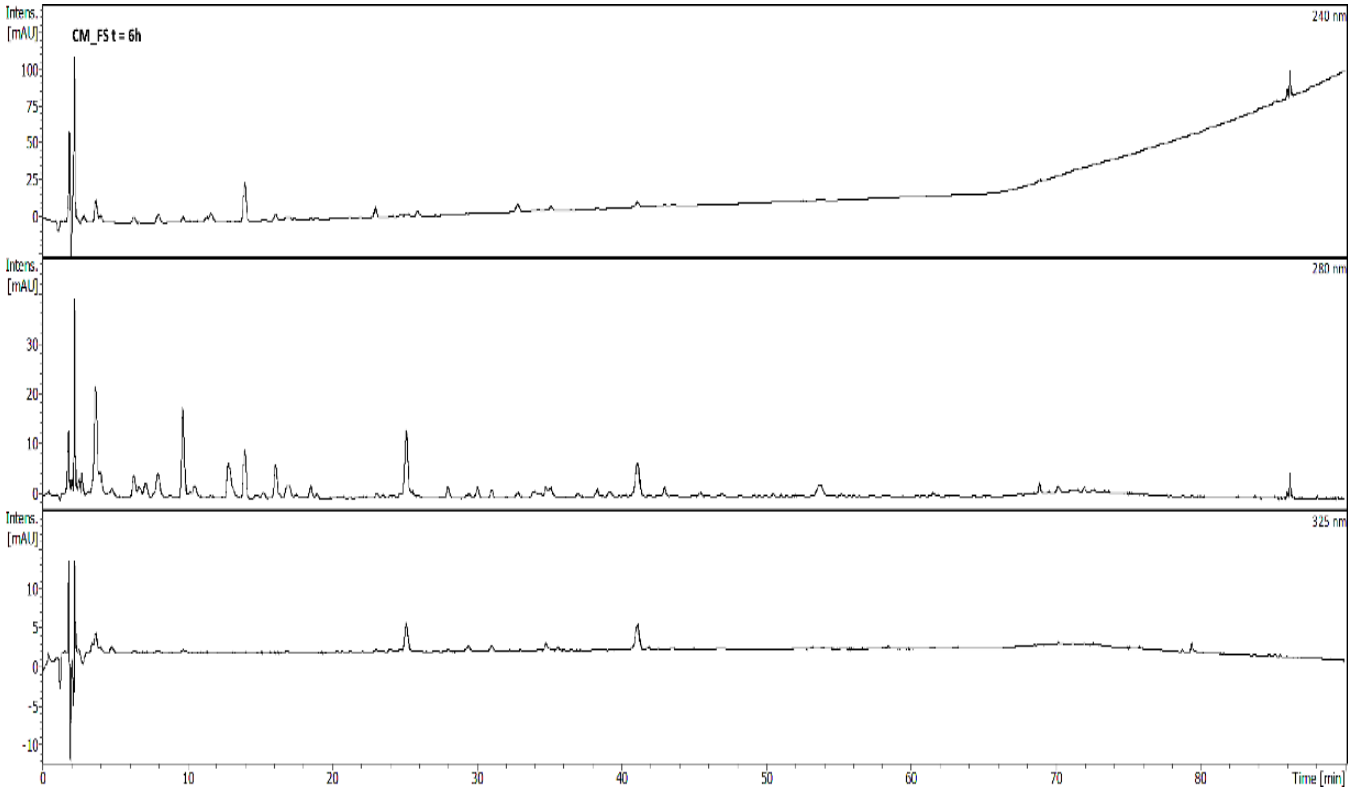

Figure S4. HPLC chromatograms of ethanolic-aqueous extract from fruits of *C. mas* treated with FS in t = 6 h registered at 240, 280, and 325 nm.

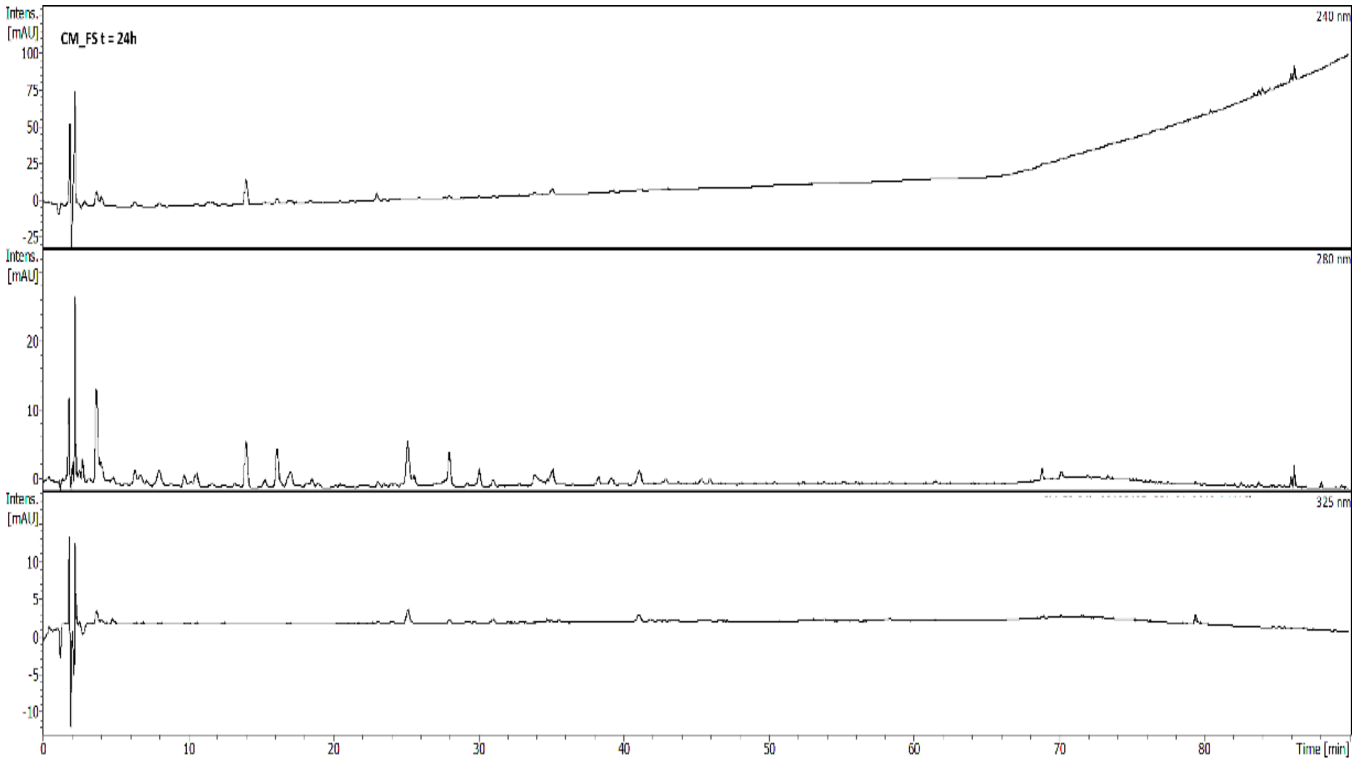

Figure S5. HPLC chromatograms of ethanolic-aqueous extract from fruits of *C. mas* treated with FS in t = 24 h registered at 240, 280, and 325 nm.
